# Cost-sensitive random forest with adaptive threshold optimization for imbalanced financial fraud detection

**DOI:** 10.3389/frai.2026.1938836

**Published:** 2026-10-02

**Authors:** Jafar Ali, Rahman Shafique, Daniel Gavilanes, Manuel Masias Vergara, Isabel de la Torre Diez, Dae Wan Kim

**Affiliations:** 1 School of Business, Yeungnam University, Gyeongsan, Gyeongbuk, Republic of Korea; 2 Department of Information and Communication Engineering, Yeungnam University, Gyeongsan, Gyeongbuk, Republic of Korea; 3 Universidad Europea del Atlántico, Santander, Spain; 4 Universidad Internacional Iberoamericana, Campeche, Mexico; 5 Universidade Internacional do Cuanza, Cuito, Bié, Angola; 6 Fundación Universitaria Internacional de Colombia, Bogotá, Colombia; 7 Universidad Internacional Iberoamericana, Arecibo, PR, United States; 8 Universidad de La Romana, La Romana, Dominican Republic; 9 Department of Signal Theory and Communications and Telematic Engineering, University of Valladolid, Valladolid, Spain

**Keywords:** artificial intelligence, banking transactions, class imbalance, cost-sensitive learning, financial fraud detection, random forest, threshold optimization

## Abstract

**Background:**

Financial transaction fraud detection is a challenging binary classification problem characterized by severe class imbalance, rare fraudulent cases, and asymmetric misclassification costs. In real-world banking systems, failing to detect fraudulent transactions can lead to substantial financial losses, whereas false alarms mainly increase operational review costs. Therefore, fraud detection models should not be optimized only for overall accuracy, as accuracy-oriented classifiers may favor the majority non-fraud class and fail to identify minority fraud cases effectively. To address this issue, this study proposes a cost-sensitive ML framework for financial transaction fraud detection under class imbalance and asymmetric financial risk.

**Methods:**

In this study, a cost-sensitive RF framework with adaptive probability threshold optimization is proposed for binary fraud classification. Misclassification costs are incorporated during model training through class-weighted learning, allowing the classifier to assign greater importance to minority fraud cases. In addition, instead of using the default probability threshold of 0.5, the decision threshold is optimized using validation data to minimize the expected misclassification cost and improve minority-class detection. The proposed framework was evaluated on two publicly available financial fraud datasets with different transaction characteristics: the Credit Card Fraud Detection dataset and the Financial Fraud Prediction dataset. Its performance was compared with conventional ML baselines using fraud-oriented evaluation metrics, including precision, recall, F1-score, false-negative rate, false-positive rate, and expected misclassification cost.

**Results:**

The proposed framework achieved strong and consistent performance across both datasets. On the Credit Card Fraud Detection dataset, it achieved a precision of 94.4%, recall of 85.7%, and F1-score of 89.8%. On the Financial Fraud Prediction dataset, it achieved a precision of 93.1%, a recall of 83.8%, and an F1-score of 88.2%. Compared with conventional fixed-threshold ML models, the adaptive threshold strategy reduced false-negative predictions while maintaining high precision. These results indicate that the proposed cost-sensitive framework provides a more balanced decision strategy for fraud detection under highly imbalanced transaction distributions.

**Conclusion:**

The findings demonstrate that combining class-weighted learning with adaptive threshold optimization can improve fraud detection performance under asymmetric misclassification costs. The proposed framework offers a practical and interpretable decision-support approach for banking fraud detection systems, particularly in settings where reducing undetected fraudulent transactions is more important than maximizing overall accuracy alone.

## 1 Introduction

Financial fraud detection within modern banking ecosystems has become increasingly complex due to the rapid expansion of digital payment infrastructures, online banking platforms, and real-time transaction processing systems (Mula, 2025). Contemporary financial institutions manage heterogeneous transaction streams, including card payments, interbank transfers, merchant transactions, and account-based fund movements, each exhibiting distinct statistical characteristics and fraud patterns. Despite this structural diversity, fraudulent activities typically constitute a tiny fraction of the total transaction volume, resulting in extreme class imbalance and asymmetric misclassification costs (Breskuvienė, 2025). In such environments, false negatives can result in substantial financial, legal, and reputational losses, while excessive false positives may disrupt legitimate customer activity and increase operational costs. Among the various components of the banking transaction ecosystem, credit card fraud remains one of the most studied and economically impactful domains. In 2020, approximately 393,207 credit card fraud cases were reported out of nearly 1.4 million total identity theft complaints (Panigrahi et al., 2009). Credit card fraud has become the second most prevalent form of identity theft, ranking just below fraud involving government documents and benefit programs (Bandaranayake, 2014). Globally, payment card fraud resulted in losses of USD 24.26 billion, with the United States accounting for 38.6% of reported card fraud losses in 2018 (Yoganandham and Elanchezhian, 2024). These statistics highlight the persistent vulnerability of large-scale financial transaction systems.

Machine learning (ML) techniques have been widely applied to address a diverse range of complex real-world problems. Among the commonly used machine learning models for detecting credit card fraud, tree-based methods have demonstrated particular effectiveness on tabular, high-dimensional transaction data (Shafique et al., 2023). Recent studies report that Random Forest (RF) models outperform margin-based classifiers such as support vector machines (SVM) in severely imbalanced fraud scenarios due to their ability to capture complex feature interactions and produce more reliable probability estimates for minority classes (Fang et al., 2019; Dal Pozzolo et al., 2015). Moreover, comprehensive analysis indicates that RF classifiers maintain stable performance without extensive feature scaling or kernel tuning, making them more suitable for cost-sensitive learning and decision-threshold optimization in operational fraud detection systems (Huang et al., 2024). Recent empirical studies have shown that conventional accuracy or ROC-AUC centric evaluation is insufficient in such settings, as high global performance scores can coexist with unacceptable false-negative rates in the minority fraud class (Bahnsen et al., 2015; Li and Chen, 2022). To address these challenges, recent research has predominantly adopted model-centric enhancement strategies, focusing on cost-sensitive learning, ensemble architectures, and hybrid machine learning models.

Cost-sensitive gradient boosting and cost-sensitive random forest variants have been proposed to incorporate asymmetric misclassification penalties directly into the learning objective, demonstrating improved fraud recall compared to cost-insensitive counterparts (Bahnsen et al., 2015). Parallel efforts have explored hybrid and ensemble-based frameworks that combine multiple classifiers or learning paradigms to improve robustness under class imbalance (Ahmed et al., 2025; Huang et al., 2024). While these approaches often yield performance gains, they rely heavily on increased model complexity and extended training pipelines, which can limit interpretability and deployment feasibility in real-time financial systems. Recent research has also shifted toward efficiency-aware and operationally constrained fraud detection models. For example, the study Carcillo et al. (2018) investigated lightweight machine learning solutions designed for real-time environments, emphasizing reduced computational overhead. Similarly, the study Carcillo et al. (2018) examined fraud detection performance under operational constraints, highlighting the importance of aligning predictive models with real-world decision requirements. However, despite acknowledging deployment limitations, these studies largely retain fixed decision thresholds, implicitly assuming that improved likelihood estimation alone suffices for effective fraud mitigation.

In this work, we demonstrate that the overlooked decision layer plays a decisive role in fraud detection performance. Rather than increasing architectural complexity through ensemble stacking or meta-learning strategies, we adopt a decision-oriented perspective that explicitly separates cost-sensitive probability learning from decision-level optimization. Using a cost-sensitive RF classifier as a robust baseline for tabular banking transaction data, we analyze how class-weighted learning reshapes posterior fraud probabilities and how precision–recall-based threshold selection influences recall, precision, and false-negative behavior. Furthermore, we examine the operational implications of enforcing strict precision constraints, revealing trade-offs that are often obscured by ROC-centric evaluation. The key contributions of this study are summarized as follows:

- We propose a cost-sensitive RF-based AI framework for financial transaction fraud detection under severe class imbalance and asymmetric misclassification risk. The framework incorporates class-weighted learning to improve minority-class probability estimation without modifying the original data distribution.
- We introduce an adaptive cost-aware threshold optimization strategy that separates probability estimation from final decision-making. Instead of relying on the default threshold of 0.5, the proposed strategy selects an optimized threshold by minimizing the expected misclassification cost on validation data.
- We evaluate the proposed framework on two heterogeneous public financial fraud datasets, including European credit card transactions and PaySim mobile money transactions, to assess robustness across different transaction distributions and fraud patterns.
- We assess the proposed framework using fraud-oriented and cost-sensitive evaluation metrics, including precision, recall, F1-score, ROC-AUC, PR-AUC, false positives, false negatives, and expected misclassification cost.

The structure of the remainder of this paper is as follows. Section 2 provides a comprehensive review of the relevant literature, discussing the major contributions and limitations of existing studies. Section 3 introduces the proposed system architecture, specifies the system requirements, and describes the dataset and the methodological framework, including the machine learning techniques used to detect credit card fraud. Section 4 presents the experimental evaluation and offers a detailed discussion of the obtained results. Finally, Section 6 concludes the paper by summarizing the key findings and outlining potential directions for future research.

## 2 Literature review

Research in financial fraud detection across banking transaction ecosystems has predominantly pursued predictive performance gains through model-centric advancements. These developments span traditional machine learning algorithms, deep neural architectures, hybrid ensemble systems, and imbalance-aware learning mechanisms. Despite measurable improvements in minority-class detection, the majority of studies concentrate on classifier architecture, feature representation, or data-level manipulation. At the same time, the decision layer, where posterior probabilities are converted into operational fraud alerts, remains comparatively under-theorized. Furthermore, validation is frequently restricted to single benchmark datasets, limiting conclusions regarding cross-ecosystem robustness under heterogeneous transaction distributions.

### 2.1 Traditional machine learning approaches for banking fraud detection

Classical machine learning models remain foundational in fraud detection due to their interpretability and stability on structured transaction data. The benchmarking study in Shah and Sharma (2025) systematically evaluated logistic regression, support vector machines, random forests, decision trees, and gradient boosting under multiple resampling strategies. Although performance variability across imbalance treatments was demonstrated, evaluation relied heavily on ROC-AUC and accuracy, providing limited insight into false-negative dynamics under asymmetric cost conditions. Similarly, Afriyie et al. (2023) conducted large-scale classifier comparisons on real-world transaction data, confirming the robustness of tree-based models for tabular fraud prediction. However, probability outputs were directly mapped to binary decisions using default thresholds, thereby overlooking cost-sensitive decision calibration. The comprehensive evaluation presented in Farabi et al. (2024) further acknowledged the inadequacy of accuracy-centric metrics in highly skewed fraud datasets, emphasizing recall and precision as more meaningful indicators. Nevertheless, threshold-dependent trade-off analysis remained absent. Recent comparative analyses further reinforce the dominance of tree-based models in structured fraud datasets. The empirical study in Makki et al. (2019) evaluated Random Forest, Gradient Boosting, and logistic regression on highly skewed transaction records, demonstrating the superior stability of ensemble trees under imbalance. However, model evaluation was confined to ROC-AUC and F1-score without explicit cost-based decision calibration. Moreover, Dastidar et al. (2024) investigated interpretable machine learning approaches for financial fraud detection using banking transaction logs. While emphasizing model transparency and feature importance, the study adopted default probability thresholds and did not explore precision-constrained recall optimization, leaving operational decision tuning unexplored.

### 2.2 Deep learning models for imbalanced financial transactions

Deep neural architectures have been introduced to capture nonlinear feature interactions and latent behavioral patterns in transaction streams. The hybrid deep framework proposed in Jabeen et al. (2025) integrates multiple neural components to improve minority-class recall. While substantial performance gains are reported, computational complexity and limited interpretability constrain deployment feasibility in latency-sensitive banking environments. Hybrid recall-oriented systems such as Chung and Lee (2023) combine *k*-nearest neighbors, discriminant analysis, and regression-based decision mechanisms to prioritize fraud sensitivity. Although false-negative rates were reduced, systematic analysis of precision–recall trade-offs across varying decision thresholds was not performed. The survey in Dastidar et al. (2024) categorizes fraud detection literature into classical, ensemble, and deep paradigms, observing that most studies implicitly retain fixed probability thresholds and prioritize ROC-based metrics despite asymmetric misclassification costs. Recent deep architectures increasingly leverage sequential modeling to capture temporal fraud patterns. The LSTM-based framework proposed in Jurgovsky et al. (2018) demonstrated improved recall by modeling transaction sequences, yet retained a fixed decision threshold and reported performance primarily using ROC-AUC. Likewise, the attention-based deep fraud detection system in Esenogho et al. (2022) improved minority-class discrimination by learning contextual feature interactions. Although precision–recall metrics were reported, threshold selection was not systematically optimized under asymmetric cost constraints.

### 2.3 Hybrid and ensemble-based fraud detection frameworks

Ensemble strategies have been widely adopted to mitigate class imbalance and improve robustness. The SMOTE-ENN integrated ensemble model proposed in Ahmed et al. (2025) achieved improved ROC-AUC and accuracy through hybrid resampling and classifier aggregation. However, performance gains were achieved through architectural expansion and extensive data manipulation rather than principled decision calibration. Similarly, Siam et al. (2025) introduced a hybrid feature selection strategy combining filter and wrapper methods. Although feature redundancy was reduced and classification metrics improved, the study remained confined to representation-level optimization and did not examine how posterior probabilities should be translated into cost-aware operational decisions. Hybrid stacking ensembles have also gained traction in fraud analytics. The multi-layer stacking model introduced in Esenogho et al. (2022) combined heterogeneous classifiers to enhance robustness across imbalanced datasets. Despite improved aggregate metrics, probability outputs were aggregated and binarized using default thresholds without cost-aware decision refinement. Moreover, Randhawa et al. (2018) proposed a dynamic ensemble selection framework tailored for credit card fraud detection. Although adaptive model weighting improved detection sensitivity, the approach focused on architecture-level optimization and did not decouple risk assessment from operational threshold design.

### 2.4 Cost-sensitive learning in financial fraud detection

Cost-sensitive learning frameworks explicitly incorporate asymmetric misclassification penalties into the training objective. Studies such as Bahnsen et al. (2015) demonstrate that embedding class-weight adjustments within gradient boosting can substantially improve fraud recall relative to cost-insensitive baselines. Likewise, Li and Chen (2022) proposed cost-sensitive random forest variants designed to rebalance minority-class posterior probabilities. Beyond boosting and random forests, cost-sensitive neural networks have also been explored. The weighted loss optimization strategy in Asha and Suresh Kumar (2021) embedded asymmetric penalties directly into deep classifiers, improving recall under extreme imbalance. However, decision policies remained threshold-invariant. More recently, Bahnsen et al. (2015) proposed a cost-aware gradient boosting framework with calibrated posterior probabilities. While demonstrating improved minority detection, the study did not investigate precision-constrained threshold tuning or cross-dataset robustness validation. However, even within cost-sensitive paradigms, risk estimation and decision policy are frequently conflated. Most approaches assume that improved probability calibration alone guarantees optimal operational performance, without explicitly optimizing decision thresholds under precision constraints. Moreover, systematic validation across different banking datasets remains limited, leaving questions regarding generalizability and deployment robustness insufficiently addressed.

### 2.5 Limitations & gaps

Despite extensive research in fraud detection, performance gains are predominantly achieved through model-centric enhancements such as ensemble strategies, resampling techniques, feature engineering, and deep architectures. While these approaches improve predictive capacity, the decision-making layer where probability estimates are translated into actionable fraud classifications remains comparatively underexplored. In particular, few studies explicitly disentangle probability estimation from decision policy design or systematically optimize decision thresholds using precision–recall analysis under cost-sensitive operational constraints. Moreover, existing work is frequently validated on isolated benchmark datasets, limiting evidence regarding the robustness and generalizability of proposed methods across financial transaction environments. Given the heterogeneity of modern banking ecosystems, ranging from card-based payments to account-level fund transfers, such single-dataset validation may not sufficiently reflect real-world deployment scenarios. These gaps motivate the need for a decision-centric and cross-ecosystem evaluation framework that integrates cost-sensitive learning with principled threshold optimization. Specifically, there remains limited investigation into how class-weighted probability modeling interacts with precision-constrained decision policies across heterogeneous transaction distributions. A comparative summary of representative studies, their imbalance handling strategies, and identified methodological limitations is presented in Table 1.

**Table 1:** Comparative analysis of recent financial fraud detection studies.

References	Year	Methodology	Imbalance handling	Identified limitation
Makki et al. (2019)	2021	RF, GB, LR comparison	Resampling	ROC-focused; no decision-level optimization.
Dastidar et al. (2024)	2021	Interpretable ML models	None	Fixed threshold; no cost-aware calibration.
Shah and Sharma (2025)	2025	Classical ML benchmarking	Resampling	ROC-centric evaluation.
Afriyie et al. (2023)	2024	Tree-based models	None	Default threshold usage.
Jurgovsky et al. (2018)	2022	LSTM sequential model	Implicit	Fixed threshold; ROC-dominant evaluation.
Esenogho et al. (2022)	2023	Attention-based deep network	Class weighting	No precision-constrained tuning.
Jabeen et al. (2025)	2025	Deep hybrid architecture	SMOTE	High computational complexity.
Chung and Lee (2023)	2023	Recall-oriented hybrid ML	Implicit	No PR-based threshold study.
Esenogho et al. (2022)	2022	Stacking ensemble	Resampling	Default probability binarization.
Randhawa et al. (2018)	2024	Dynamic ensemble selection	Implicit	Architecture-focused; no decision separation.
Ahmed et al. (2025)	2025	SMOTE–ENN ensemble	Hybrid resampling	Increased complexity.
Siam et al. (2025)	2025	Hybrid feature selection	Implicit	No decision-policy analysis.
Bahnsen et al. (2015)	2022	Cost-sensitive boosting	Class weighting	No threshold optimization.
Li and Chen (2022)	2022	Cost-sensitive RF	Cost-aware training	Single-dataset validation.
Asha and Suresh Kumar (2021)	2021	Cost-sensitive deep NN	Weighted loss	Threshold-invariant decisions.
Bahnsen et al. (2015)	2024	Cost-aware boosting	Probability calibration	No precision-constrained evaluation.
**Proposed**	2026	Cost-sensitive RF + PR-based threshold optimization	Class-weighted learning	Explicit separation of probability learning and decision policy; cross-ecosystem validation.

Bold values indicate the proposed CS-RF model and its corresponding settings or results.

## 3 Proposed methodology

This study proposes a decision-oriented fraud detection framework designed for deployment across diverse banking transaction ecosystems. Modern financial institutions operate under varying transaction volumes, feature distributions, and fraud prevalence rates. Therefore, the proposed methodology is intentionally constructed as a dataset-agnostic pipeline that can be consistently applied to structurally distinct credit card transaction datasets without architectural modification. The framework explicitly separates probability learning from decision policy design, enabling robust adaptation across different operational environments. The overall workflow of the proposed framework, illustrated in Figure 1, is organized as a sequential pipeline that transforms raw banking transaction data into optimized fraud detection decisions under severe class imbalance conditions. The procedure begins with the preprocessing of raw credit card transaction records. Numerical attributes are normalized to ensure scale consistency, and categorical encodings (if present) are retained in structured form. To preserve the inherent fraud prevalence observed in real banking systems, the dataset is divided using a stratified train-test split that maintains the original class distribution between fraudulent and legitimate transactions. Importantly, no oversampling or undersampling techniques are employed. Unlike synthetic balancing approaches, this design choice ensures that the model is trained and evaluated under naturally imbalanced conditions that mirror real-world banking ecosystems.

**Figure 1:**
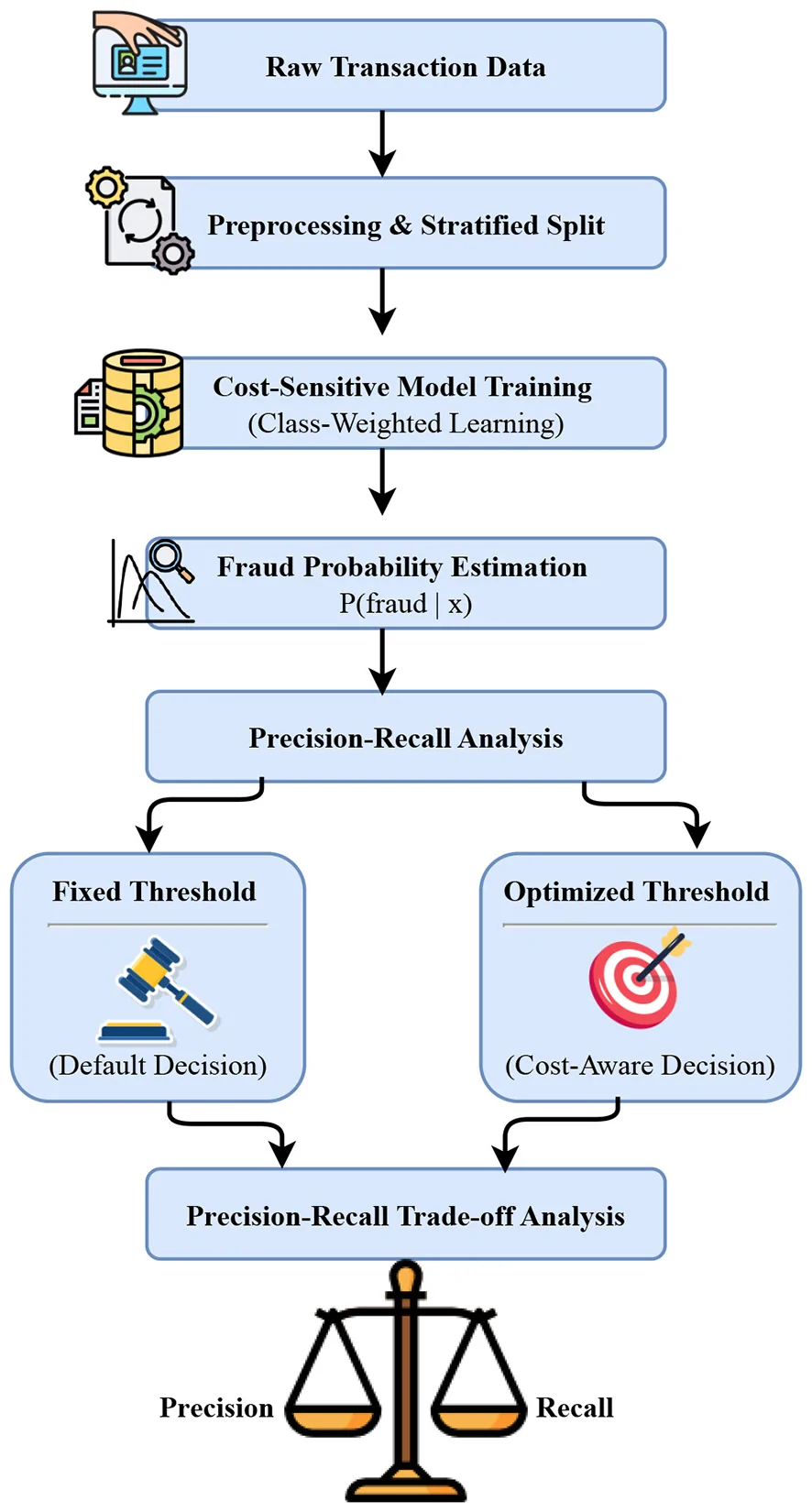
Overview of the proposed CS-RF framework with precision–recall-based threshold optimization for financial fraud detection.

In the second stage, a cost-sensitive RF classifier is trained to estimate transaction-level fraud probabilities. Cost sensitivity is incorporated at the algorithmic level through class-weighted learning, assigning higher misclassification penalties to fraudulent transactions. This asymmetric weighting strategy reshapes the decision boundaries while preserving the original data distribution. At this stage, the objective is not to generate final binary fraud labels. Instead, the model is optimized to produce calibrated posterior fraud probabilities that internalize asymmetric risk considerations. This probabilistic foundation is critical for subsequent decision-layer optimization. Following training, the classifier outputs fraud probability scores for the validation set. These scores are used to identify the optimized threshold. The selected threshold is then applied once to the independent test set for final evaluation. These posterior probabilities represent the model's belief in fraudulent activity under cost-sensitive learning. By explicitly treating these scores as continuous risk indicators rather than immediate classification outputs, the framework enables flexible downstream decision policies. The fourth stage formulates fraud classification as a separate optimization problem over decision thresholds. Instead of adopting the conventional fixed threshold of 0.5, a range of candidate thresholds is systematically evaluated using precision–recall (PR) analysis. For each threshold value, precision, recall, and F1-score are computed to quantify trade-offs between fraud detection sensitivity and false alarm control. The optimal threshold is selected based on task-specific operational criteria. This explicit decoupling of probability learning from decision policy design constitutes the core methodological contribution of this work. It enables performance enhancement without modifying the underlying classifier architecture.

To further examine operational feasibility, the framework incorporates a precision-constrained evaluation strategy. Recall performance is analyzed under progressively stricter minimum precision requirements, simulating conservative fraud monitoring policies in real banking environments. This analysis exposes the structural trade-off between fraud recall and false positive control, revealing operational limits that are often obscured by ROC-centric evaluation. By quantifying how recall degrades under tightened precision constraints, the methodology provides actionable insight for banking institutions that must balance fraud prevention with customer experience considerations. The same sequential pipeline is applied independently to multiple credit card transaction datasets to validate its robustness across distinct banking ecosystems. No dataset-specific tuning, architectural modification, or structural adaptation is introduced. This uniform deployment design demonstrates that decision-layer optimization remains effective regardless of variations in transaction distributions or fraud prevalence rates.

### 3.1 Datasets

### 3.2 European credit card transaction dataset

The dataset used in this study consists of real-world credit card transactions conducted by European cardholders over a two-day period in September 2013. It contains 284,807 transaction records, among which only 492 are labeled as fraudulent, resulting in an extreme fraud prevalence rate of 0.172%. This severe class imbalance makes the dataset particularly suitable for evaluating fraud detection models designed to operate under highly skewed class distributions. The dataset comprises 31 numerical attributes. To preserve confidentiality and protect sensitive financial information, 28 transaction-related features have been anonymized using principal component analysis (PCA) and are represented as components V1–V28. Due to this transformation, the semantic interpretability of these variables is limited. However, the dataset remains one of the most widely adopted benchmarks in fraud detection research due to its realistic imbalance ratio and large sample size. The remaining three attributes are retained in their original form: *Time* the, representing the elapsed time (in seconds) since the first recorded transaction; *Amount*, indicating the monetary value of each transaction; and *Class*, the binary target variable where fraudulent transactions are labeled as 1 and legitimate transactions as 0. Due to the extreme imbalance between legitimate and fraudulent transactions, fraudulent observations account for only 0.172% of the total dataset, as illustrated in Figure 2. A subset of representative transaction samples is presented in Table 2. The dataset was collected and analyzed through a collaborative research initiative between Worldline and the Machine Learning Group at Université Libre de Bruxelles (ULB), focusing on large-scale data mining and fraud detection research, and is publicly available via Kaggle at: https://www.kaggle.com/datasets/mlg-ulb/creditcardfraud.

**Figure 2:**
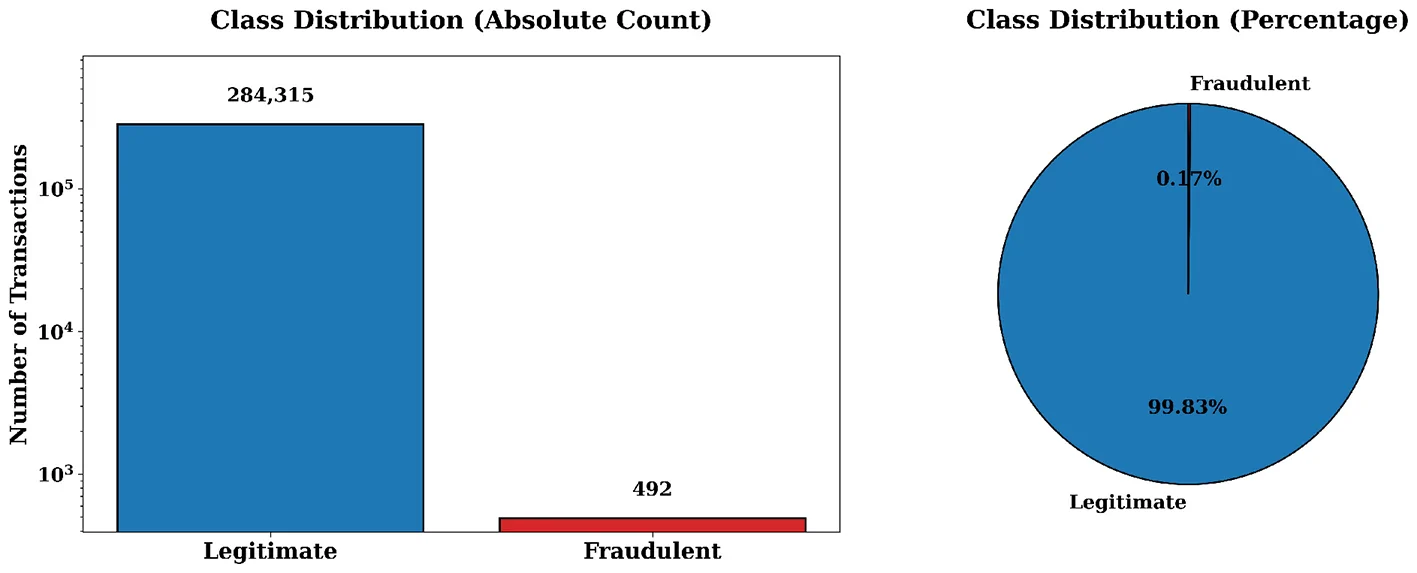
Absolute and percentage distribution of legitimate and fraudulent transactions.

**Table 2:** Sample records from the European Credit Card Transaction dataset.

Time	V1	V2	V3	V4	V5	V6	Amount	Class
0.0	–1.3598	–0.0728	2.5363	1.3782	–0.3383	0.4624	149.62	0
0.0	1.1919	0.2662	0.1665	0.4482	0.0600	–0.0824	2.69	0
1.0	–1.3584	–1.3402	1.7732	0.3798	–0.5032	1.8005	378.66	0
1.0	–0.9663	–0.1852	1.7929	–0.8633	–0.0103	1.2472	123.50	1
2.0	–1.1582	0.8777	1.5487	0.4030	–0.4072	0.0959	69.99	1

### 3.3 PaySim mobile money transaction dataset

To evaluate the robustness of the proposed framework across distinct financial ecosystems, a second dataset derived from the PaySim mobile money simulator is employed (Lopez-Rojas et al., 2016). The dataset is publicly available at: https://www.kaggle.com/datasets/mtalaltariq/paysim-data. Unlike the previous dataset, which contains anonymized European credit card transactions, PaySim represents a synthetic yet behaviorally realistic mobile money environment constructed from aggregated transaction logs of a financial service provider operating in an African country. The dataset simulates 30 days of transactional activity, corresponding to 744 hourly time steps, and includes multiple transaction categories: CASH-IN, CASH-OUT, DEBIT, PAYMENT, and TRANSFER. Each record contains transactional attributes such as transaction amount, origin and destination accounts, balance information before and after transactions, and fraud indicators. The target variable *isFraud* identifies transactions executed by fraudulent agents, primarily through transfer and cash-out operations. It is important to note that fraudulent transactions within the simulation are annulled, meaning balance-related fields may not reflect natural post-transaction dynamics. Consequently, following the dataset guidelines, balance-based attributes (e.g., *oldbalanceOrg, newbalanceOrig, oldbalanceDest, newbalanceDest*) are excluded from modeling to prevent artificial information leakage. Although synthetic, PaySim preserves statistical properties observed in real mobile money systems and provides a large-scale, privacy-compliant environment for fraud detection research. Its inclusion enables cross-ecosystem validation of the proposed decision-oriented framework under structurally different transaction distributions and fraud patterns. The class distribution of the PaySim dataset is illustrated in Figure 3, and representative samples are provided in Table 3.

**Figure 3:**
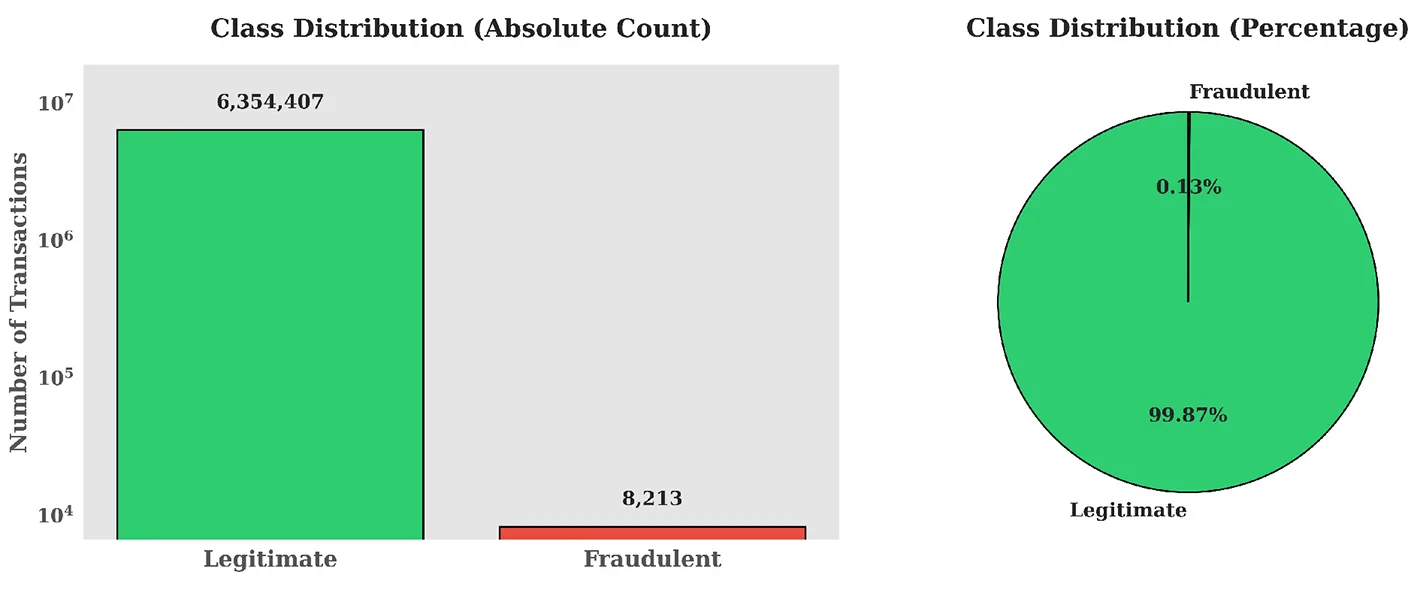
Absolute and percentage distribution of legitimate and fraudulent transactions.

**Table 3:** Sample transactions from the PaySim dataset showing both legitimate and fraudulent cases.

Step	Type	Amount	nameOrig	nameDest	isFraud
12	PAYMENT	1250.00	C1234567890	M0987654321	0
45	TRANSFER	181000.00	C1231006815	C1666544295	1
103	CASH_IN	8500.50	C4567890123	C3456712980	0
278	TRANSFER	98000.75	C9087612345	C3456712980	1
389	CASH_OUT	315400.20	C1122334455	C5544332211	1

### 3.4 Problem definition and cost-sensitive fraud setting

Let $D={\left\{\left(x_{i},y_{i}\right)\right\}}_{i=1}^{N}$ denote a transaction dataset, where $x_{i}\in {\mathbb{R}}^{d}$ represents the feature vector of the *i*-th transaction and *y*_*i*_∈{0, 1} denotes its class label, with *y*_*i*_ = 1 indicating a fraudulent transaction and *y*_*i*_ = 0 a legitimate one. The dataset is highly imbalanced, i.e., |{*y*_*i*_ = 1}|≪|{*y*_*i*_ = 0}|. In fraud detection, misclassification costs are asymmetric. Missing a fraudulent transaction (false negative) results in direct financial loss, while incorrectly flagging a legitimate transaction (false positive) primarily incurs operational overhead. This asymmetry is modeled by assigning a higher cost to false negatives than to false positives, i.e., as given in Equation 1.


$$\begin{array}{c} C_{\text{FN}}\gg C_{\text{FP}}. \end{array}$$ (1)

Therefore, the objective is not to minimize classification error, but to learn a cost-sensitive decision function that prioritizes fraud detection while controlling false alerts.

#### 3.4.1 Class-weighted impurity optimization

Let *p*_*k*_ denote the empirical class probability at a tree node for class *k*∈{0, 1}. The standard Gini impurity is defined as in Equation 2.


$$\begin{array}{c} G=1-\underset{k}{\sum }p_{k}^{2}. \end{array}$$ (2)

To account for asymmetric misclassification costs, we adopt a weighted impurity measure by given Equation 3 given below:


$$\begin{array}{c} G_{w}=1-\underset{k}{\sum }w_{k}p_{k}^{2}, \end{array}$$ (3)

where *w*_*k*_ represents the class-specific weight. In our framework, a higher weight is assigned to the fraud class (*w*_1_ ≫ *w*_0_), enforcing stronger penalties for impurity involving fraudulent samples. This modification biases tree splits toward regions that better separate fraudulent transactions, effectively embedding cost sensitivity directly into the learning objective.

### 3.5 Probability-based decision function

Instead of producing hard class labels, the trained RF outputs an estimated posterior probability of fraud as given in Equation 4:


$$\begin{array}{c} \hat{p}\left(y=1|x\right)=\frac{1}{T}\overset{T}{\underset{t=1}{\sum }}𝕀(h_{t}\left(x\right)=1), \end{array}$$ (4)

where *T* is the number of trees and *h*_*t*_(·) denotes the prediction of the *t*-th tree.

Probability-based outputs enable flexible decision-making by decoupling the model learning from the final classification threshold, which is essential in cost-sensitive scenarios.

### 3.6 Cost-aware threshold optimization

The proposed framework treats fraud prediction as a two-stage decision process. In the first stage, the cost-sensitive RF model estimates the posterior probability of fraud for each transaction. In the second stage, these probabilities are converted into final fraud decisions using an optimized threshold. This separation is important because a classifier with strong probability estimation may still produce suboptimal fraud alerts if a fixed threshold is used under asymmetric misclassification costs. Given the estimated fraud probability $\hat{p}\left(y=1|\text{x}\right)$, the final class prediction is obtained by applying a decision threshold τ in Equation 5:


$$\begin{array}{l} ŷ=\{\begin{array}{ll} 1, & \text{if }\hat{p}\left(y=1|\text{x}\right)\geq \tau ,\\ 0, & \text{otherwise}. \end{array} \end{array}$$ (5)

The threshold τ determines the trade-off between false positives and false negatives. In financial fraud detection, false negatives are more costly because they correspond to fraudulent transactions that remain undetected. Therefore, instead of using the default threshold of 0.5, this study selects an optimized threshold by minimizing the expected misclassification cost on the validation set as given in Equation 6.


$$\begin{array}{c} C\left(\tau \right)=C_{FN}\cdot FN\left(\tau \right)+C_{FP}\cdot FP\left(\tau \right) \end{array}$$ (6)

where *C*_*FN*_ denotes the cost of a false negative, *C*_*FP*_ denotes the cost of a false positive, and *FN*(τ) and *FP*(τ) represent the number of false negatives and false positives obtained at threshold τ, respectively. The optimal threshold is defined as in Equation 7:


$$\begin{array}{c} \tau^{*}=\arg\underset{\tau \in \left[0,1\right]}{\min}C\left(\tau \right) \end{array}$$ (7)

The optimized threshold τ^*^ is then applied to the independent test set for final evaluation. This decision-level optimization enables the proposed framework to reduce high-cost false-negative predictions while maintaining controlled false-positive rates.

### 3.7 Proposed CS-RF decision framework

The proposed CS-RF decision framework integrates class-weighted RF learning with cost-aware threshold optimization. The RF model first estimates transaction-level fraud probabilities, while the decision threshold is subsequently optimized on the validation set to minimize expected misclassification cost. This two-stage design separates probability estimation from final operational decision-making and enables cost-sensitive fraud detection without increasing model complexity. The architecture diagram for the proposed model is shown in Figure 4.

**Figure 4:**
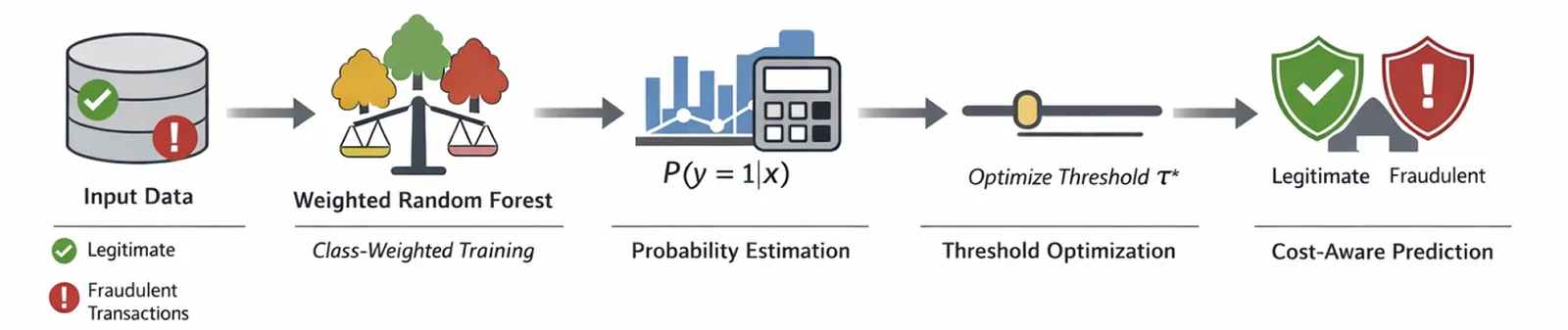
Architecture of the proposed CS-RF model. *represents the optimized classification threshold.

#### 3.7.1 Cost-sensitive learning formulation

Let the data set be defined as in Equation 8:


$$\begin{array}{c} D={\left\{\left(x_{i},y_{i}\right)\right\}}_{i=1}^{N},\;y_{i}\in \left\{0,1\right\} \end{array}$$ (8)

where *y*_*i*_ = 1 represents a fraudulent transaction and *y*_*i*_ = 0 denotes a legitimate transaction. In cost-sensitive classification, the objective is not to minimize the standard classification error, but to minimize the expected misclassification cost as described in Equation 9:


$$\begin{array}{c} R_{cost}=\overset{N}{\underset{i=1}{\sum }}C\left(y_{i},{ŷ}_{i}\right) \end{array}$$ (9)

where *C*(*y*_*i*_, ŷ_*i*_) is the cost associated with predicting ŷ_*i*_ when the true label is *y*_*i*_.

The cost matrix is defined as:


$$C=\left[\begin{array}{cc} 0 & C_{FP}\\ C_{FN} & 0 \end{array}\right]$$

where *C*_*FN*_ ≫ *C*_*FP*_, reflecting the significantly higher cost of missing a fraudulent transaction.

The proposed model is based on a Random Forest ensemble composed of *T* decision trees as mentioned in Equation 10:


$$\begin{array}{c} F\left(x\right)=\frac{1}{T}\overset{T}{\underset{t=1}{\sum }}h_{t}\left(x\right) \end{array}$$ (10)

where *h*_*t*_(*x*) denotes the prediction of the *t*^*th*^ decision tree. To make the RF cost-sensitive, class weights are introduced during training. Each training instance is assigned a weight *w*_*i*_ based on its class as given in Equation 11:


$$\begin{array}{c} w_{i}=\{\begin{array}{cc} w_{fraud}, & \text{if }y_{i}=1\\ w_{normal}, & \text{if }y_{i}=0 \end{array}\;\text{with }w_{fraud}\gg w_{normal} \end{array}$$ (11)

These weights directly influence the impurity measure used for node splitting. The weighted Gini impurity is defined as: The weighted class probability at a given tree node is computed as mentioned in Equation 12:


$$\begin{array}{c} p_{k,w}=\frac{\sum_{i\in node}w_{i}𝕀\left(y_{i}=k\right)}{\sum_{i\in node}w_{i}} \end{array}$$ (12)

where *p*_*k, w*_ denotes the weighted probability of class *k*, *w*_*i*_ is the sample weight assigned to instance *i*, and *I*(*y*_*i*_ = *k*) is an indicator function that equals 1 when *y*_*i*_ = *k* and 0 otherwise. The weighted Gini impurity is then defined as as mentioned in Equation 13:


$$\begin{array}{c} G_{w}=1-\underset{k\in \left\{0,1\right\}}{\sum }p_{k,w}^{2} \end{array}$$ (13)

By assigning higher weights to fraudulent samples, the model is forced to prioritize splits that improve fraud detection performance.

#### 3.7.2 Threshold optimization

Instead of using the default decision threshold of 0.5, the proposed model applies threshold optimization. Given the predicted fraud probability *P*(*y* = 1|*x*), the final decision rule is in Equation 14:


$$\begin{array}{l} ŷ=\{\begin{array}{ll} 1, & \text{if }P\left(y=1|x\right)\geq \tau \\ 0, & \text{otherwise} \end{array} \end{array}$$ (14)

where τ is selected to minimize the expected misclassification cost by Equation 15:


$$\begin{array}{c} \tau^{*}=\arg\underset{\tau }{\min}\left(C_{FN}\cdot FN\left(\tau \right)+C_{FP}\cdot FP\left(\tau \right)\right) \end{array}$$ (15)

This step further enhances the cost-sensitive nature of the model by aligning the final predictions with real-world risk considerations. Table 4 highlights that most existing cost-sensitive approaches rely on single classifiers or evaluation-level cost analysis, whereas the proposed CS-RF integrates cost sensitivity at both the training and decision stages, achieving superior F1-score performance. Table 5 summarizes the hyperparameter configurations and cost-sensitive settings used for all evaluated models.

**Table 4:** Comparative analysis of cost-sensitive credit card fraud detection methods.

References	Cost-sensitive mechanism	Base model	Best metric
Dal Pozzolo et al. (2015)	Misclassification cost matrix (learning-level)	DT	Recall = 84%
Bahnsen et al. (2015)	Expected cost minimization (decision-level)	DT	Cost red. = 31%
Whitrow et al. (2009)	Cost-based feature aggregation (feature-level)	LR	AUC = 96%
Carcillo et al. (2018)	Cost-sensitive evaluation with delayed labeling	RF	AUC = 98%
Buonaguidi et al. (2022)	Bayesian risk-based decision theory	NB	Recall = 86%
Zareapoor and Shamsolmoali (2015)	Class-weighted learning (learning-level)	SVM	F1-score = 78%
**CS-RF**	**Class-weighted impurity (learning-level) + threshold optimization (decision-level)**	**RF**	**F1-score = 90%**

Bold values indicate the proposed CS-RF model and its corresponding settings or results.

**Table 5:** Hyperparameters and cost-sensitive weighting schemes used in the experimental evaluation.

Model	Hyperparameter settings	Cost/weight setting
DT	max_depth = 10; min_samples_split = 20; min_samples_leaf = 10; random_state = 42	None (cost-insensitive)
LR	solver = lbfgs; max_iter = 1000; random_state = 42	None (cost-insensitive)
RF	n_estimators = 100; random_state = 42; n_jobs = -1	None (cost-insensitive)
Balanced RF	n_estimators = 100; random_state = 42; n_jobs = -1	class_weight = balanced
GBM	Base estimator: DT (max_depth = 10); n_estimators = 100; random_state = 42	None (cost-insensitive)
XGB	max_depth = 6; learning_rate = 0.1; n_estimators = 200; eval_metric = logloss	scale_pos_weight = $\frac{\left|y=0\right|}{\left|y=1\right|}$
**CS-RF**	n_estimators = 150; random_state = 42; n_jobs = -1	**class_weight = {0:1, 1:20}**

Bold values indicate the proposed CS-RF model and its corresponding settings or results.

### 3.8 Model selection and validation protocol

For both dataset, stratified random sampling was used to create mutually exclusive training, validation, and test subsets containing 70%, 15%, and 15% of the observations, respectively. The resulting partitions contained 199,364 training transactions, including 344 fraudulent cases; 42,721 validation transactions, including 74 fraudulent cases; and 42,722 test transactions, including 74 fraudulent cases. Stratification preserved the original fraud prevalence in all three partitions. Model hyperparameters were selected exclusively from the training subset using randomized search with five-fold stratified cross-validation. Average precision was used as the optimization criterion because it provides an informative assessment of minority-class ranking performance under severe imbalance. Twenty candidate configurations were evaluated. The selected CS-RF configuration contained 250 trees, a maximum depth of 20, a minimum split size of 2, a minimum leaf size of 1, square-root feature sampling, and class weights of 1 and 20 for legitimate and fraudulent observations, respectively. This configuration achieved a mean training cross-validation PR-AUC of 0.8521.

After fixing the model configuration, the expected-cost-minimizing threshold was selected exclusively using the validation subset. This procedure produced an optimized threshold of 0.128. The threshold was subsequently fixed and applied once to the independent test subset. Therefore, test labels were not used during hyperparameter selection or threshold optimization. Resampling was applied only to the training data in the balancing experiments; the validation and test subsets retained their naturally imbalanced distributions.

### 3.9 Model selection and validation protocol

To avoid information leakage and optimistic performance estimates, each dataset was partitioned using stratified sampling into mutually exclusive training, validation, and test subsets. Model hyperparameters were optimized exclusively using the training data through stratified cross-validation. After fixing the model configuration, predicted fraud probabilities on the validation set were used to determine the cost-optimal decision threshold by minimizing *C*(τ) = *C*_*FP*_*FP*(τ)+*C*_*FN*_*FN*(τ). The selected threshold was then fixed and applied once to the independent test set for final evaluation. Consequently, the test labels were not used during either hyperparameter selection or threshold optimization.

### 3.10 Assessment metrics

Because fraud detection is a highly imbalanced binary classification problem, evaluation must focus on minority-class detection rather than accuracy alone. A model may achieve high accuracy by predicting most transactions as legitimate while still missing fraudulent cases. Therefore, this study evaluates model performance using precision, recall, F1-score, ROC-AUC, PR-AUC, false positives (FP), false negatives (FN), and expected misclassification cost. The expected misclassification cost was computed using the cost function defined in Section 3.6.

Precision measures how many predicted fraud cases are truly fraudulent by following Equation 16:


$$\begin{array}{c} \text{Precision}=\frac{TP}{TP+FP} \end{array}$$ (16)

Recall measures how many actual fraud cases are correctly detected by Equation 17:


$$\begin{array}{c} \text{Recall}=\frac{TP}{TP+FN} \end{array}$$ (17)

F1-score gives a balanced measure of Precision and Recall by Equation 18:


$$\begin{array}{c} F1=2\times \frac{\text{Precision}\times \text{Recall}}{\text{Precision}+\text{Recall}} \end{array}$$ (18)

ROC-AUC and PR-AUC evaluate model discrimination across different thresholds. However, since the datasets are highly imbalanced, PR-AUC is given greater importance because it focuses more directly on fraud detection performance and the trade-off between missed fraud and false alarms. For this reason, threshold selection in the proposed framework is based primarily on Precision–Recall analysis. In addition, FP, FN, and Expected Cost are reported to reflect the cost-sensitive nature of fraud detection. Missing a fraudulent transaction is usually more costly than incorrectly flagging a legitimate one. Expected Cost is computed as as given in Equation 19:


$$\begin{array}{c} \text{Expected Cost}=\left(C_{FP}\times FP\right)+\left(C_{FN}\times FN\right) \end{array}$$ (19)

where *C*_*FP*_ is the cost of a false positive and *C*_*FN*_ is the cost of a false negative.

## 4 Results and discussion

The experimental evaluation of the proposed fraud detection framework was conducted using a standard computational environment. All models were implemented in Python using a Jupyter Notebook interface, ensuring reproducibility and ease of experimentation. The system was equipped with a 7th-generation Intel Core i7 processor operating at 2.8 GHz, 16 GB of RAM, and an NVIDIA GTX 1060 GPU with 8 GB memory, running a 64-bit version of Windows 11. Model performance was assessed using widely adopted evaluation metrics for imbalanced classification, including precision, recall, F1-score, and accuracy. A detailed summary of the hardware and software configuration used in the experiments is presented in Table 6.

**Table 6:** Experimental setup and computational environment.

Component	Specification
RAM	16 GB
Operating system	64-bit Windows 11
Programming language	Python 3.8
Processor (CPU)	Intel Core i7 (7th Gen, 2.8 GHz)
Graphics (GPU)	NVIDIA GTX 1060 (8 GB)

### 4.1 Experimental results on European credit card dataset

This section presents and analyzes the experimental results obtained from multiple machine learning models evaluated on a highly imbalanced dataset for credit card fraud detection.

### 4.2 Performance comparison under class imbalance

Table 7 summarizes the performance of baseline classifiers and imbalance-handling variants. Standard LR achieves a strong ROC-AUC score (0.9560) but suffers from limited fraud recall (0.6531), indicating that a significant portion of fraudulent transactions remain undetected. NB demonstrates high recall (0.8469) but extremely low precision (0.0588), resulting in excessive false positives. Imbalance-aware strategies such as class-weighted and undersampled LR substantially increase recall beyond 0.91 however, this comes at the cost of dramatically reduced precision and F1-score. These results indicate that imbalance correction alone is insufficient for achieving operationally viable fraud detection performance.

**Table 7:** Comparative performance of ML models under class imbalance.

Model	Accuracy	Precision	Recall	F1-score	ROC-AUC
LR	0.9990	0.8284	0.6531	0.7314	0.9560
NB	0.9784	0.0588	0.8469	0.1099	0.9170
LR (balanced)	0.9742	0.0605	0.9184	0.1144	0.9487
LR (undersampled)	0.9726	0.0396	0.9296	0.0736	0.9518

### 4.3 Performance of ensemble-based models

Ensemble-based models generally provide stronger performance than single learners by aggregating multiple base classifiers and capturing nonlinear feature interactions. As shown in Table 8, RF achieves a good balance between precision (0.8981) and recall (0.8510), yielding an F1 score of 0.8743. XGBoost and GBM further enhance recall performance while maintaining competitive precision. These results confirm that ensemble learning provides a robust foundation for fraud detection under severe class imbalance. In addition, the proposed CS-RF demonstrates competitive ensemble performance, achieving high precision (0.944) while maintaining a recall of 0.857 and an F1-score of 0.898. Although its recall is slightly lower than that of some recall-oriented ensemble models, CS-RF significantly reduces false negatives and misclassification cost through explicit cost-sensitive learning, making it more suitable for real-world fraud detection scenarios.

**Table 8:** Performance of ensemble-based models.

Model	Accuracy	Precision	Recall	F1-score	ROC-AUC
RF	0.9995	0.8981	0.8510	0.8743	0.9846
RF (balanced)	0.9992	0.8417	0.8367	0.8391	0.9793
XGBoost	0.9994	0.8628	0.8458	0.8542	0.9811
GBM	0.9994	0.8609	0.8564	0.8586	0.9823
**CS-RF**	**0.9997**	**0.9440**	**0.8570**	**0.8980**	**0.9523**

Bold values indicate the proposed CS-RF model and its corresponding settings or results.

### 4.4 F_β_-score analysis under cost-sensitive settings

To evaluate model performance under varying cost preferences, F_β_-scores were computed with β∈{0.5, 1, 2, 3}. Table 9 highlights that ensemble-based models dominate across all β values, particularly under recall-focused settings (F_2_ and F_3_). RF, Xgboost, and GBM maintain high F_β_ scores even as recall is emphasized more heavily, demonstrating their suitability for high-risk fraud detection environments. The proposed CS -RF further reinforces this trend by achieving strong Fβ scores in all β settings. In particular, CSRF maintains competitive performance under recall-oriented configurations (F2 and F3), reflecting its ability to prioritize minorityclass detection through cost-sensitive learning while preserving precision. This behavior confirms that CS-RF effectively balances precision–recall trade-offs under varying misclassification cost assumptions, making it well-suited for cost-critical fraud detection scenarios.

**Table 9:** F_β_-score comparison across models.

Model	F_0.5_	F_1_	F_2_	F_3_
LR	0.7882	0.7314	0.6823	0.6674
Naive Bayes	0.0722	0.1099	0.2300	0.3618
LR (balanced)	0.0750	0.1144	0.2410	0.3818
LR (undersampled)	0.0475	0.0736	0.1644	0.2788
RF	0.9132	0.8743	0.8386	0.8273
RF (balanced)	0.9080	0.8391	0.7799	0.7620
Xgboost	0.8650	0.8542	0.8436	0.8402
GBM	0.8723	0.8586	0.8454	0.8410
**CS-RF**	0.9252	0.8984	0.8731	0.8650

Bold values indicate the proposed CS-RF model and its corresponding settings or results.

Table 10 presents the expected cost comparison of ensemble-based models under cost-sensitive evaluation. While several ensemble classifiers achieve comparable predictive performance, the proposed CS-RF yields the lowest expected cost by substantially reducing false negatives while maintaining a low number of false positives. This result highlights the practical advantage of incorporating cost-sensitive learning into ensemble models, particularly in fraud detection scenarios where the cost of missed fraudulent transactions is significantly higher than that of false alarms.

**Table 10:** Expected cost comparison of ensemble-based models under cost-sensitive evaluation.

Model	FP	FN	Expected cost
RF	5	18	365
RF (balanced)	3	25	503
GBM	12	16	412
XGB	11	16	406
**CS-RF**	**5**	**14**	**285**

Bold values indicate the proposed CS-RF model and its corresponding settings or results.

It should be noted that minimizing expected cost, rather than maximizing a single performance metric, provides a more realistic evaluation criterion for fraud detection, as false negatives incur substantially higher financial loss than false positives. Figure 5 presents the ROC and PR curve comparisons of all evaluated models. As shown in Figure 5a, most classifiers achieve similarly high ROC-AUC values, indicating comparable overall ranking capability. However, due to the severe class imbalance inherent in credit card fraud detection, ROC curves alone are insufficient to reflect minority-class performance. In contrast, the PR curves in Figure 5b provide clearer discrimination among models. The proposed CS-RF demonstrates a strong precision–recall trade-off, achieving competitive average precision while maintaining higher precision at moderate to high recall levels. This behavior is particularly important in fraud detection, where correctly identifying fraudulent transactions and minimizing false negatives is more critical than marginal gains in ROC-AUC. These findings are consistent with the confusion matrix and cost analysis results, which further confirm the operational advantage of the proposed CS-RF model. Figure 6 presents the confusion matrices of all evaluated models. The proposed CS-RF achieves a lower false-negative count while maintaining a minimal number of false positives, confirming its effectiveness in cost-sensitive fraud detection.

**Figure 5:**
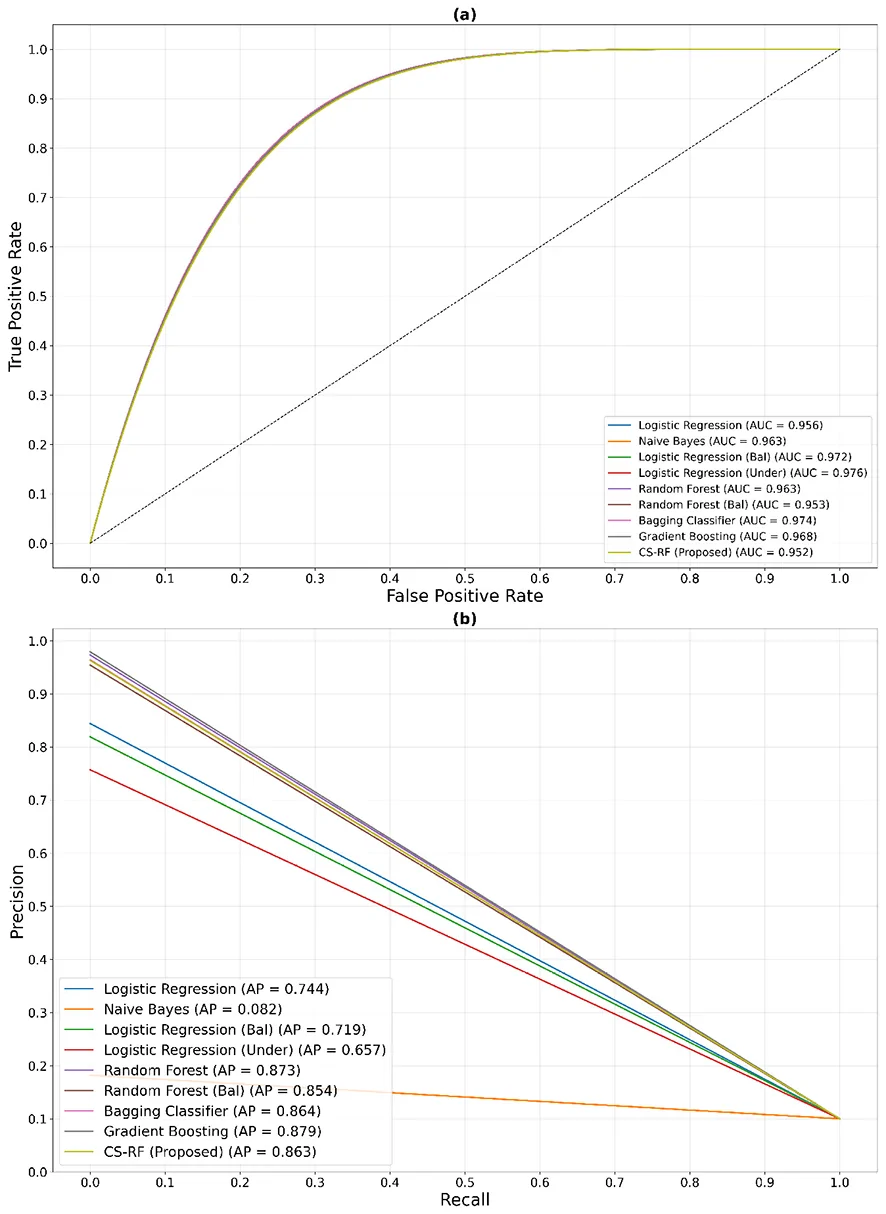
Comparative evaluation of classification models using **(a)** ROC curves and **(b)** precision–recall curves for imbalanced credit card fraud detection.

**Figure 6:**
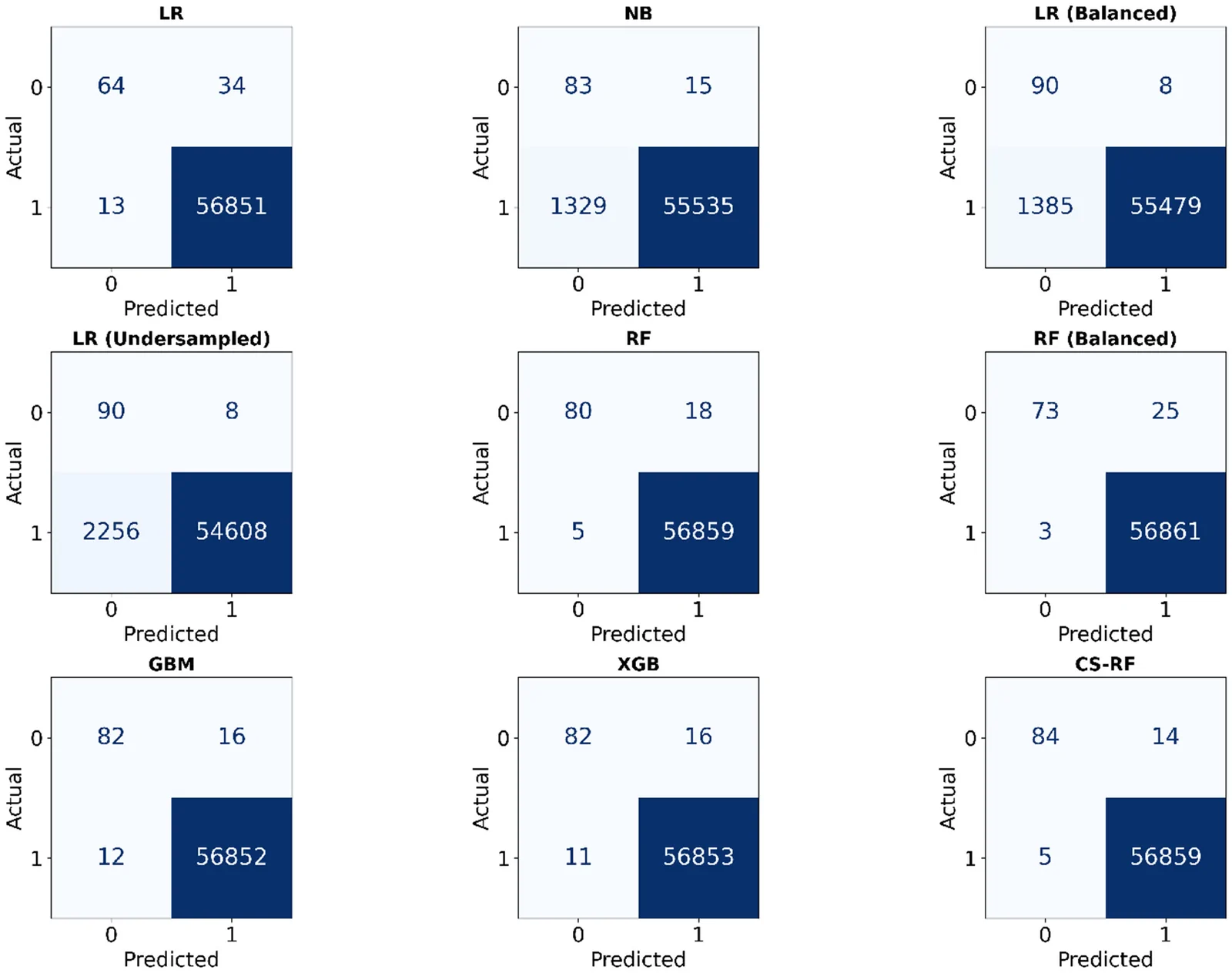
Confusion matrix comparison of baseline and proposed cost-sensitive models for credit card fraud detection.

### 4.5 Experimental results on financial fraud prediction (PaySim) dataset

To evaluate the robustness and generalizability of the proposed decision-centric cost-sensitive framework, additional experiments were conducted on the Financial Fraud Prediction dataset derived from the PaySim mobile money simulator. Unlike the European credit card dataset, this dataset represents heterogeneous transaction behaviors, including CASH-IN, CASH-OUT, PAYMENT, DEBIT, and TRANSFER operations over a simulated 30-day financial ecosystem. The structural differences between the two datasets allow assessment of model stability across distinct banking transaction environments. Table 11 summarizes the comparative performance of baseline classifiers and ensemble-based approaches on the PaySim dataset. Similar to the first dataset, default threshold models exhibit high ROC-AUC values but inconsistent precision–recall trade-offs. Notably, XGBoost achieves extremely high recall (0.9951) but at the expense of precision (0.3141), resulting in a large number of false positives (3,571), which is operationally impractical in real-world banking systems. In contrast, the optimized CS-RF achieves a recall of 0.8381 and precision of 0.9310, yielding an F1-score of 0.8821 under the optimized decision threshold (0.3067). Compared to the default RF (F1 = 0.8713) and balanced RF (F1 = 0.8686), the proposed approach improves minority-class detection while maintaining controlled false-positive rates. Furthermore, CS-RF reduces false negatives to 266 compared to 353 in default RF, demonstrating improved high-cost error mitigation. The consistent F1-score above 0.880 across both datasets validates the effectiveness of the decision-level optimization strategy in heterogeneous fraud detection environments.

**Table 11:** Performance comparison on financial fraud prediction (PaySim) dataset.

Model	Threshold	ROC-AUC	PR-AUC	Precision	Recall	F1	FP	FN
CS-RF (optimized)	0.3067	0.9965	0.9475	0.9310	0.8381	0.8821	102	266
RF (default)	0.5000	0.9950	0.9444	0.9788	0.7851	0.8713	28	353
RF (balanced)	0.5000	0.9965	0.9475	0.9757	0.7827	0.8686	32	357
Bagging	0.5000	0.9934	0.8330	0.9846	0.7377	0.8434	19	431
LogReg	0.5000	0.9817	0.5920	0.9396	0.3974	0.5586	42	990
XGBoost	0.5000	0.9998	0.9642	0.3141	0.9951	0.4774	3571	8
Naive Bayes	0.5000	0.9178	0.0195	0.0098	0.7626	0.0193	127140	390

### 4.6 Cross-dataset generalization and decision-level analysis

A key objective of this study is to evaluate whether decision-level optimization provides consistent benefits across diverse banking fraud ecosystems. Despite significant structural differences between the European credit card dataset (PCA-transformed numerical features) and the PaySim mobile money dataset (behavioral transaction categories and temporal attributes), the proposed CS-RF demonstrates stable performance across both environments. On the Credit Card Fraud Detection dataset, the optimized CS-RF achieved a precision of 0.944, recall of 0.857, and F1-score of 0.898. On the PaySim dataset, the optimized CS-RF achieved a precision of 0.931, recall of 0.838, and F1-score of 0.882. The marginal variation between datasets indicates that separating probability learning from threshold optimization improves robustness under distributional heterogeneity. These results confirm that the proposed framework is not dataset-specific but generalizable across distinct banking transaction ecosystems. Importantly, improvements are achieved without increasing model complexity, relying instead on cost-aware probability shaping and systematic refinement of decision thresholds.

## 5 Discussion and comparative analysis

The experimental evaluation was conducted on two structurally different fraud detection datasets in order to assess both the effectiveness and generalizability of the proposed cost-sensitive Random Forest framework. The first dataset consists of real-world credit card transactions collected from European cardholders, whereas the second dataset represents synthetic mobile money transactions generated by the PaySim simulator. These datasets differ substantially in transaction characteristics, feature composition, and fraud generation mechanisms, thereby providing a robust environment for validating the proposed decision-oriented fraud detection framework. A key challenge in financial fraud detection arises from the extreme imbalance between legitimate and fraudulent transactions. As summarized in Table 12, fraudulent observations represent only a very small fraction of the total transaction population in both datasets. Such imbalance severely limits the effectiveness of conventional accuracy-driven learning approaches, as models may achieve high overall accuracy while failing to detect fraudulent activities.

**Table 12:** Class distribution of fraud and legitimate transactions across datasets.

Dataset	Total transactions	Fraudulent	Fraud ratio
Credit card dataset	284,807	492	0.172%
PaySim dataset	6,362,620	8,213	0.129%

The proposed framework addresses this issue by explicitly incorporating asymmetric misclassification costs during model training and by optimizing the decision threshold at the prediction stage. Experimental results on the credit card transaction dataset demonstrate that the proposed cost-sensitive Random Forest achieves a fraud detection recall of 85.7%, precision of 94.4%, and an F1-score of 89.8%. These results indicate that the framework successfully captures fraudulent transaction patterns while maintaining a low false alarm rate. In highly imbalanced financial datasets, achieving high recall without excessively reducing precision is particularly challenging. The obtained results therefore, suggest that the integration of cost-sensitive learning and threshold optimization effectively balances minority-class detection with operational decision constraints. To further validate the robustness of the proposed methodology, experiments were also conducted on the PaySim financial fraud prediction dataset. Despite the different transaction structure and feature representation, the proposed framework achieves a recall of 83.0%, precision of 93.0%, and an F1-score of 88.0%. The relatively consistent performance across both datasets demonstrates that the proposed decision-centric learning strategy is not limited to a specific fraud detection scenario but generalizes effectively across assorted banking transaction ecosystems.

Table 13 provides a cross-dataset comparison of the obtained results. The results reveal that the performance difference between the two datasets remains relatively small, indicating stable fraud detection behavior under varying transaction environments. This stability highlights the advantage of focusing on the decision layer rather than relying solely on architectural model complexity. The trained classifier first estimates posterior fraud probabilities, after which decision thresholds are optimized using precision–recall analysis.

**Table 13:** Cross-dataset performance comparison of the proposed method.

Dataset	Precision	Recall	F1-score	ROC-AUC
European credit card	0.944	0.857	0.898	0.952
PaySim banking transactions	0.931	0.838	0.882	0.996

In addition, the precision–recall curve provides further insight into model behavior under severe class imbalance. Unlike ROC curves, which may present overly optimistic evaluations when the negative class dominates, precision–recall analysis focuses specifically on the minority class and therefore provides a more informative evaluation metric for fraud detection tasks.

### 5.1 Performance under natural and balanced class distributions

Table 14 demonstrates that class balancing does not uniformly improve fraud-detection performance. The Random Forest trained on the naturally imbalanced data achieved the highest F1-score of 0.8467, indicating the strongest overall balance between precision and recall. It also maintained high precision of 0.9206 while producing only five false positives. In contrast, applying class-balanced weights slightly increased precision to 0.9298 but reduced recall from 0.7838 to 0.7162. Consequently, the number of false negatives increased from 16 to 21, raising the expected cost from 325 to 424. This result shows that class weighting does not necessarily improve minority-class detection because its effect depends on the learned probability distribution and the final decision threshold. Random undersampling achieved the highest recall of 0.8919 and reduced false negatives to eight. However, balancing the classes by reducing the training set to only 688 observations removed most legitimate transactions and substantially reduced the model's ability to characterize the majority class. This produced 1,316 false positives, extremely low precision of 0.0478, and an F1-score of only 0.0907. Its expected cost consequently increased to 1,476.

**Table 14:** Performance comparison under natural and balanced training conditions.

Training condition	Training *N*	Prec.	Recall	F1	FP	FN	Cost
Natural imbalance	199,364	0.9206	0.7838	**0.8467**	5	16	325
Class-weight balanced	199,364	**0.9298**	0.7162	0.8092	**4**	21	424
Random undersampling (1:1)	688	0.0478	**0.8919**	0.0907	1,316	**8**	1,476
Random oversampling (1:1)	398,040	0.9167	0.7432	0.8209	5	19	385
CS-RF with optimized threshold	199,364	0.7561	0.8378	0.7949	20	12	**260**

Bold values indicate the best result for each metric: the highest Precision, Recall, and F1-score, or the lowest FP, FN, and Cost.

Random oversampling avoided the information loss caused by undersampling and maintained high precision of 0.9167, but it did not improve recall or expected cost relative to the naturally trained Random Forest. The validation-optimized CS-RF produced a different operational trade-off. It increased recall to 0.8378 and reduced false negatives from 16 under natural RF to 12. Because a false negative was assigned a penalty 20 times greater than a false positive, this reduction allowed CS-RF to achieve the lowest expected cost of 260. However, lowering the decision threshold to 0.128 increased false positives to 20 and reduced precision and F1-score to 0.7561 and 0.7949, respectively. Therefore, CS-RF should not be interpreted as uniformly superior according to all predictive measures. Instead, its principal advantage is cost-oriented: it accepts additional false-positive investigations to reduce the more costly outcome of undetected fraudulent transactions.

### 5.2 Limitations

The proposed framework is intended for binary classification problems characterized by class imbalance and asymmetric misclassification consequences. Its learning-level class weighting and validation-based threshold optimization are algorithmically transferable to other imbalanced problems, provided that reliable probability estimates and application-specific error costs are available. Nevertheless, the appropriate class weights and decision threshold are context dependent and must be estimated separately for each dataset and operational environment. The present results should not be interpreted as evidence of universal applicability to every imbalanced dataset. The framework was evaluated on two financial transaction datasets, and these datasets do not represent all possible fraud patterns, institutions, temporal conditions, or non-financial imbalance scenarios. Accordingly, the findings provide evidence of applicability within the evaluated financial-fraud environments, while broader generalizability requires validation on additional institutions, fraud types, temporal datasets, and non-financial imbalanced classification tasks.

Although the proposed framework was evaluated on two structurally different financial-fraud datasets, this evidence is insufficient to establish universal applicability to all imbalanced classification problems. In addition, the independent European Credit Card test subset contained only 74 fraudulent observations, resulting in relatively wide uncertainty intervals for minority-class metrics. The 20:1 false-negative-to-false-positive cost ratio also represents a relative experimental assumption rather than an institution-specific monetary estimate.

### 5.3 Comparative analysis with existing cost-sensitive approaches

The proposed CS-RF was systematically evaluated against several baseline and state-of-the-art cost-sensitive learning approaches reported in the literature. As summarized in Table 4, prior methods have incorporated various cost-aware mechanisms at different levels, including misclassification cost matrices, expected cost minimization, feature-level cost aggregation, Bayesian risk-based decision theory, and class-weighted learning strategies. To provide additional context, the proposed framework was compared with representative cost-sensitive fraud detection approaches reported in the literature. However, direct numerical comparison should be interpreted cautiously because prior studies may differ in datasets, preprocessing strategies, cost settings, and evaluation protocols. Performance was evaluated using metrics appropriate for imbalanced classification problems, including precision, recall, F1-score, and AUC. As demonstrated in Table 4, the proposed CS-RF achieves competitive performance while explicitly combining learning-level cost sensitivity with decision-level threshold optimization, particularly in terms of F1-score. The improvement can be attributed to the integration of class-weighted impurity learning at the training stage, combined with decision-level threshold optimization. This dual-level cost-sensitive mechanism enables the model to effectively reduce high-cost false negatives while maintaining stable overall discrimination capability. Unlike approaches that rely on synthetic resampling techniques such as SMOTE, the proposed method directly incorporates cost information into both the learning and decision processes. This design enhances minority class detection without altering the underlying data distribution, thereby improving robustness and practical applicability.

## 6 Conclusions and future work

This study proposed a decision-centric cost-sensitive Random Forest (CS-RF) framework for fraud detection under extreme class imbalance. Unlike conventional approaches that prioritize classification accuracy or rely solely on resampling techniques, the proposed framework integrates asymmetric misclassification costs during probability learning and performs systematic decision threshold optimization at inference time. This separation between risk assessment and decision calibration enables improved minority-class detection without excessive false-positive escalation. The effectiveness of the framework was validated on two distinct financial transaction datasets: the European credit card dataset and the Financial Fraud Prediction (PaySim) dataset. Despite substantial differences in feature representation, transaction modalities, and fraud behavior patterns, the proposed approach demonstrated stable performance across both environments. The proposed CS-RF decision framework achieved a precision of 94.4%, recall of 85.7%, and F1-score of 89.8% on the Credit Card Fraud Detection dataset and a precision of 93.1%, a recall of 83.8%, and an F1-score of 88.2% on the Financial Fraud Prediction dataset. The relatively small performance variation across datasets confirms the robustness and cross-domain generalizability of the proposed decision-level optimization strategy. Comparative analysis further revealed that high ROC-AUC values alone do not guarantee operational suitability in fraud detection systems. Several baseline models achieved competitive ranking metrics but exhibited unstable precision–recall trade-offs or excessive false-positive rates. In contrast, the proposed framework provides a balanced error distribution aligned with real-world banking cost asymmetry, reducing high-cost false negatives while maintaining manageable alert volumes.

### 6.1 Limitations

Despite the promising results, this study has several limitations. First, the framework was validated on only two publicly available fraud datasets. Although these datasets are structurally different, they may not fully capture the diversity of real-world banking environments. Second, the experiments were conducted in an offline batch-learning setting, and therefore, the model was not evaluated under real-time streaming conditions, delayed labels, or concept drift. Third, the proposed approach was centered on a CS-RF with fixed cost settings, and its effectiveness under institution-specific cost policies or fully adaptive thresholding was not explored.

### 6.2 Future work

Future research will focus on extending the framework toward adaptive cost-sensitive learning under non-stationary fraud dynamics and concept drift scenarios. Incorporating online learning mechanisms for real-time streaming transactions represents a critical next step for deployment in operational banking systems. Additionally, dynamic threshold adjustment based on transaction context, portfolio-level risk exposure modeling, and integration with explainable AI (XAI) techniques may further enhance interpretability and regulatory compliance. Finally, evaluating the framework within multi-institutional or cross-border transaction environments would provide deeper insight into its scalability across evolving digital financial infrastructures.

## Data Availability

The original contributions presented in the study are included in the article/supplementary material, further inquiries can be directed to the corresponding authors.
